# 1425. "Influence of outpatient antibiotic prescriptions in prevalence of ESBL producing Enterobacteriaceae in local hospitals"

**DOI:** 10.1093/ofid/ofad500.1262

**Published:** 2023-11-27

**Authors:** Rafael Ponce, Patrick Kinsella, Darien Campbell

**Affiliations:** Mercer University School of Medicine, Macon, Georgia; Mercer University School of Medicine, Macon, Georgia; Atrium Health Navicent, Macon, Georgia

## Abstract

**Background:**

The increase in antibiotic resistance has been linked to antibiotic exposure. However, initiatives of antibiotic stewardship programs have been associated with decrease in antibiotic utilization but without a clear decrease in antibiotic resistance. We hypothesized that outpatient antibiotic exposure in communities geographically close to hospitals contribute to their incidence of ESBL producing Enterobacteriaceae (ESBL-PE).

Outpatient antibiotic prescriptions per 1000 population by zip code

**Figure:**
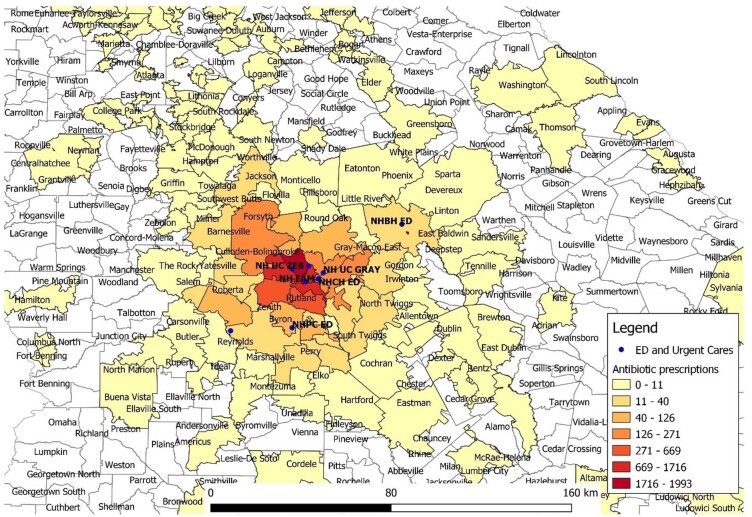

Total antibiotic prescriptions per zip code

**Figure:**
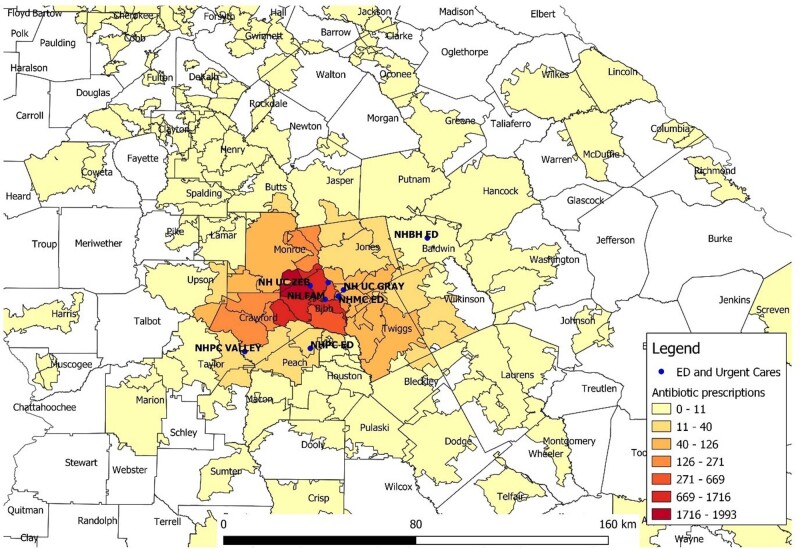

**Methods:**

From March 2022 to March 2023 we evaluated antibiotic utilization in the inpatient and outpatient setting of a healthcare system in central Georgia. Outpatient settings included urgent cares, emergency rooms, and primary care clinics. The incidence of ESBL-PE was obtained from each hospital’s antibiogram. We calculated the number of outpatient prescriptions per zip code. Nearest neighbor analysis was used to determine the closest hospital for each zip code, with each zip code having a unique "nearest" hospital. The number of antibiotic prescriptions for those zip codes were assigned to that hospital. To estimate the antibiotic “burden” for each hospital, we used days of therapy (DOT) per 1000 days present of inpatient antibiotic use, plus antibiotic prescriptions per zip code population. Percentage of ESBL-PE for each hospital was the independent variable. QGIS v3.8 was used for spatial analysis.

Inpatient antibiotic use and ESBL-PE percentage

**Figure:**
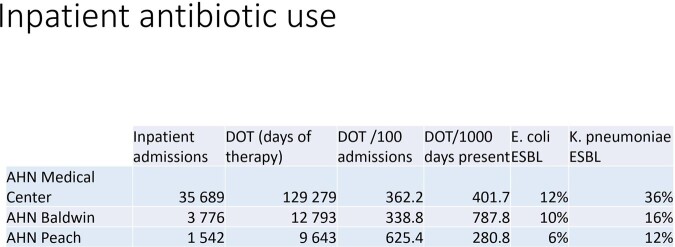

Outpatient antibiotic use

**Figure:**
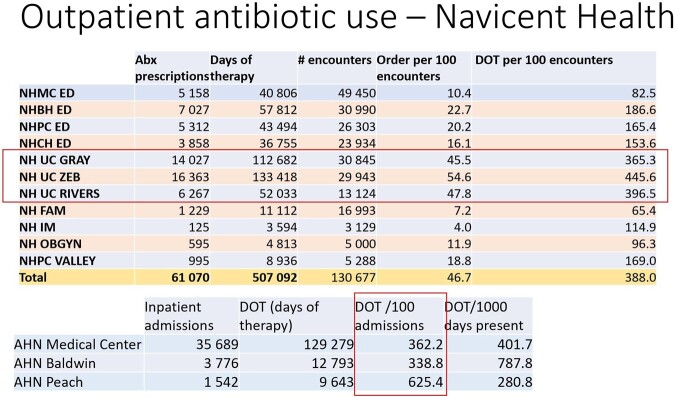

**Results:**

There were 61 070 outpatient antibiotic prescriptions and 500 092 DOT. Prescriptions were given to residents of over 1000 different zip codes of nearly 20 states. 60% of the prescriptions were from urgent care centers, 1 for every 2 encounters. Inpatient antibiotic use varied among hospitals, with no correlation between antibiotic use and percentage of ESBL-PE. Linear regression model had an R2 of over 0.8 although with a p-value of 0.8.

**Conclusion:**

The geographic reach of outpatient antibiotic prescriptions is of concern. The burden in their surrounding communities could be contributing to antibiotic resistance in hospitals, explaining the discrepancy of inpatient antibiotic use and antibiograms. Efforts to decrease antibiotic resistance should also include interventions in outpatient facilities. Our model didn’t reach statistical significance, likely due to the small hospital sample size, which we are currently evaluating.

**Disclosures:**

**All Authors**: No reported disclosures

